# Exploring the potential of a standardized test in physiotherapy: making emotion, embodiment, and therapeutic alliance count for women with chronic pelvic pain

**DOI:** 10.3389/fpsyg.2023.1166496

**Published:** 2023-08-03

**Authors:** Cathrine Maria Boge-Olsnes, Mette Bech Risør, Gunn Kristin Øberg

**Affiliations:** ^1^Department of Health and Care Sciences, Faculty of Health Sciences, UiT The Arctic University of Norway, Tromsø, Norway; ^2^Department of Public Health, University of Copenhagen, Copenhagen, Denmark; ^3^Department of Community Medicine, UiT The Arctic University of Norway, Tromsø, Norway; ^4^Department of Clinical Therapeutic Services, University Hospital North Norway, Tromsø, Norway

**Keywords:** embodiment / bodily experience, chronic pelvic pain, physiotherapy, standardized performance test, women health, enactive theory

## Abstract

**Introduction:**

There has been an increased use of standardized measurements in health care meant to provide objective information to enhance the quality and effectivity of care. Patient performance tests are based on standardized predefined criteria with a limited focus. When facing multifaceted health conditions, information expanding the predefined criteria in a standardized test may be required to understand the patient’s complex symptoms. Relying on test information based on measurements according to functional biology, one risks missing information communicated by the sensitive and expressive body of the individual patient. The aim of this article is to investigate how body, self and illness perception is constituted as a co-construction between a physiotherapist and a patient with complex symptoms, expanding the use of a standard physiotherapy test.

**Methods:**

This qualitative study is based on video-recordings and in-depth interviews of seven women with the complex health condition chronic pelvic pain. The video recordings consist of the patients performing the Standard Mensendieck test pre- and post-treatment with Norwegian psychomotor physiotherapy. The interviews are based on the patients` and the physiotherapists` conversations while watching and elaborating on these video recordings. Empirical data is analyzed within the theoretical perspectives of phenomenology and enactive theory, especially focusing on the concepts of embodiment and intersubjectivity.

**Results:**

Taking an embodied approach, considering the body as expressive, communicative, and vulnerable to the environment and context, the results show that through bodily expressions the patients experienced the test situation as demanding, thus providing information beyond what the test was intended to measure. Additionally, when administering a standardized test, the interaction between the therapist and the patient had an impact on the results. Sensitive attention towards the patients bodily expressive emotions as a vital part of the interaction, reinforced therapeutic alliance by ensuring the integrity and autonomy of the patient.

**Discussion:**

Mutual communication, gave new insights regarding the patients’ complex symptoms and reinforced their belief in themselves and their recovery processes. Applying the patient’s expertise on herself and her life together with the professional expertise may make health care an interdependent practice where sensemaking is a co-construction of meaning between the patient and the health personnel.

## Introduction

In health care, there has been a general emergence of health-outcome measurement tools meant to provide patient data to objectively demonstrate the results patients achieve due to therapeutic interventions. This information is meant for patients, clinicians, researchers, third-party payers, and health care policy administrators to enhance the quality and effectivity of care. In multifaceted health conditions, a single standardized test, which usually has limited focus, may not be sufficient to cover the complexity of the patient’s problems. To better grasp complex health challenges, the literature encourages an expanded understanding by including mental and social factors in the comprehension of the body and health (Nicholls and Gibson, 2010; Kirkengen and Thornquist, 2012; Eriksen et al., 2013).

In physiotherapy, patient performance-based tests are widely embraced, both in the clinic and in research (Brukner et al., 2017). Implementing tests in evaluating treatment outcome is considered to improve educated decisions about best practice. The performance-based tests offer information concerning a person’s ability to complete a relevant task in a controlled setting and can be used before, during or after treatment course to measure the patients progress (Beattie, 2001; Kvåle et al., 2003). One test used in physiotherapy is the Standard Mensendieck test developed by Haugstad et al. (2006) (Appendix 1), as a comprehensive body examination of patients with psychosomatic disorders. The goal of this test is to measure motor function and the effect of therapy according to quality of movements based on principles derived from functional anatomy. Herein lies the implication of a reductionistic view in physiotherapy where the functional significance of the body is reduced to the musculoskeletal system, covering mechanical properties, not including that mental and social factors affect movement. Critical voices warn that in assessments of the body as a strictly functional biological entity (Nicholls and Gibson, 2010; Richter and Maric, 2022), there is a risk that health professionals may overlook the communicative and expressive body (Thornquist, 2006).

Haugstad et al. (2006) applied the Standard Mensendieck test studying the clinical characteristics of women with chronic pelvic pain. They found a specific pattern of posture and movement in women with chronic pelvic pain deviating from asymptomatic women. The symptomatic women showed poorer coordination, stability and balance, limited movements, restricted breathing, and used a minimal area of support (Haugstad et al., 2006). The results indicate that the test may be a useful instrument in the evaluation of patients and that the therapist may use this information in treatment when working with posture and movements of daily life (Haugstad et al., 2006).

Chronic pelvic pain is a complex and multifaceted condition affecting women worldwide (Lamvu et al., 2021). Research displays multiple and complex symptoms (Ayorinde et al., 2015; Lamvu et al., 2021), and accounts of challenging life experiences like sexual abuse and difficult childhoods is a common finding in both quantitative and qualitative studies on this patient group (Lampe et al., 2003; As-Sanie et al., 2014). Therapeutic alliance, defined as the product of the patient’s and the therapist’s conscious determination and ability to work together (Bordin et al., 1994), is highlighted as especially important regarding women with chronic pelvic pain (Fougner and Haugstad, 2015; Grossnickle et al., 2019). There has been a call for conceptual clarity of therapeutic alliance within physiotherapy (Kayes and McPherson, 2012; Søndenå et al., 2020), however communication (verbal and non-verbal), including empathy, active listening and mutual understanding, is highlighted as a key determinant within the clinical encounter (Søndenå et al., 2020). As standardized tests are based on predefined assumptions and strict procedures, communication as a bi-directional sense making process is restricted (Anjum et al., 2020) and therapeutic alliance can be challenging. Research shows that the value of agreement in the therapist-patient relation is especially important in treating women with chronic pelvic pain (Fougner and Haugstad, 2015; Grossnickle et al., 2019) as they, because of multiple symptoms in intimate body parts and additional physiological and psychological distress (Ayorinde et al., 2015; Lamvu et al., 2021), are considered a particularly vulnerable patient group.

In Norway, some patients with chronic pelvic pain are treated with the specialty, Norwegian psychomotor physiotherapy. This approach bases treatment on an understanding that the mind–body entity is deeply involved in our relationship to ourselves, others, and the world (Thornquist, 2022). This insight is in accordance with the enactive approach which offers a distinctive view of how mental life relates to bodily activity (De Jaegher and Di Paolo, 2007; Fuchs and De Jaegher, 2009). Enactive theory understands the body not as an isolated and purely physical phenomenon, but as a living and sensemaking structure that is constantly in interaction with its surroundings (Sørvoll et al., 2022). Within the philosophy of phenomenology, the double constitution of embodiment points to how we can perceive our body in a twofold way: as a physical object on the one hand and a sensing subject on the other (Leder, 1990). In interaction we relate to the subjective body of the other as expressions of the other’s emotions. This makes the body a vital part of interaction, since subjects understand and move each other based on mutual interpretation of bodily expressions (Colombetti, 2014). However, in the description of the Standard Mensendieck test the body as communicative is not emphasized. Instead, the test measures exclusively the patient movements relative to predefined anatomical standards.

The aim of this article is to investigate how body, self and illness perception is constituted as a co-construction between the physiotherapist and the patient. We explore if expanding the interpretation to also include the patients sensitive and expressive body, may expand the use of the Standard Mensendieck test. This article is based on a study of women with chronic pelvic pain experiences of Norwegian psychomotor physiotherapy, using the Standard Mensendieck test as a valuation of the participants quality of movements pre- and post-treatment. Standard Mensendieck test was chosen because it takes a brief time to perform; it is already used in the evaluation of treatment for chronic pelvic pain and displays conditions in the patient that are considered important in Norwegian psychomotor physiotherapy.

## Methodology

This research project is guided and informed by the phenomenological concept of embodiment and enactive theory. The phenomenological concept of embodiment is grounded on the idea that human experience is wholly dependent on the body, which links body, mind, and surroundings together in an inseparable intertwined unity (Gallagher and Zahavi, 2012). Enactive theory builds on a synthesis of insights from different fields of science and five intertwined tenets define the enactive paradigm (De Jaegher and Di Paolo, 2007), one of which is the phenomenological concept of *embodiment.* The subject’s life is an embodied, socially and culturally embedded being-in-the-world (Merleau-Ponty, 2002), and the enactive approach explores embodiment as a significant part of sensemaking between people in interaction (Fuchs and De Jaegher, 2009). Embodiment, sense-making and intersubjectivity have been the principal concepts in our understanding of our data. We are further inspired by what Høffding and Martiny (2016) call the “phenomenological interview.” They explain this methodological framework as a two-tier process where the data collection is informed by certain phenomenological commitments which suppose that people give meaning to their experiences through interpretative processes, and that they act intentionally in relation to their environment. This understanding in turn informs a phenomenological investigation in the analysis process (Høffding and Martiny, 2016).

### Method

We used a qualitative method with a combination of in-depth interviews and video-recordings of the participants performing the Standard Mensendieck test. The main purpose of using a performance test was to investigate the participants’ experiences of the possible changes in movement quality after the treatment period. We thus needed access to the participants’ subjective experiences related to these changes which was the motivation for video-recording the test and showing the recordings to the participants while interviewing. Comparing the movement quality between the pre-and post-treatment test was the initial topic of the in-depth interviews where the purpose was to investigate how the participants understood changes.

### Research team and reflexivity

The first author is a physical therapist (PT) specialized in Norwegian psychomotor physiotherapy with extensive experience in treating women with chronic pelvic pain. As a PhD student she is in this project doing research in her own field. While doing the data collection and the transcription of the video-recordings, her professional competence gave the advantage of following up on certain topics that might have been overseen without her insights. On the other hand, her profession might have made her blind to detect and reflect on topics she found self-evident (Yardley, 2000). The second author is a professor in medical anthropology, and the third author is a professor in physiotherapy, specialized in pediatric physiotherapy. The research team’s different disciplines, unique skills, and intellectual and moral priorities helped to challenge the understanding of all research processes. The diversity of perspectives has been particularly important because of the first author/PT’s double role. The first author’s professional role shaped the data in such a way that the first author’s interaction with the participants during the data collection is part of the data we analyzed. She will hereafter be referred to by her two roles: first author/PT.

### Recruitment

A request was addressed to the professional group of Norwegian psychomotor physiotherapy therapists in Norway inviting patients to participate in our study. The inclusion criteria were adult, female patients with chronic pelvic pain symptoms. The exclusion criteria were severe addiction or psychiatric diagnosis or disease demanding other treatment, such as cancer. Five Norwegian psychomotor physiotherapy therapists responded and recruited eight patients prior to starting treatment in the period from February 2019 to February 2020. We hoped for 8–10 participants, and unfortunately, covid 19 prevented the post-treatment test of one participant which obliged us to exclude her from this study. Our study was approved by The Regional Ethical Committee REK Nord in January 2019 (approval no. REK-Nord 2018/2533). Written informed consent was obtained from all participants upon inclusion in the study.

### Participants

The seven participants, who will from now be referred to as patients, were from urban areas in northern and southern Norway. Their average age was 32 (range: 20–56). One was studying, three were working and three were on long-term sick leave. The patients had complex and overlapping symptoms in the pelvic area with pain corresponding to diagnoses such as vulvodynia, vaginismus, endometriosis, irritable bowel syndrome, dyspareunia, and hemorrhoids. They all suffered from muscular pain in many parts of their body and for some anxiety, depression and exhaustion were their main problems.

### Data collection

Video recordings of the patients performing the movement test Standard Mensendieck test is one part of the data material, supplemented with notes taken by the first author/PT (right after the video-recordings). Furthermore, the data material consists of audio recordings of the individual conversations between the patient and the first author/PT while they were watching the video recordings, which form the in-depth interviews of this study.

#### Video recording

The patients were video recorded while performing the Standard Mensendieck test before and after they were treated with Norwegian psychomotor physiotherapy for a period of approximately 6 months. Ahead of the testing, the first author/PT told the patients that the purpose was to see eventual changes in their way of moving. During the video recordings, the Standard Mensendieck test was divided into three sequences, each lasting about 1–3 min. Most of the tasks were performed facing front and then repeated facing sideways. The recordings took a maximum of 15 min to complete.

Ahead of each task sequence the first author/PT explained and demonstrated the movements as they were supposed to be performed. She then went behind the camera recording the patients’ performance, before explaining and demonstrating the next sequence. She specified that nothing should hurt and told them that she could help if they forgot what to do. The same procedure was practiced during the recordings of the pre- and post-treatment testing.

##### Description of the standard Mensendieck test tasks

###### Sequence one: standing and walking

The first test task is to be observed while standing. The patient then walks back and forth normally a couple of times, before walking the same distance faster.

###### Sequence two: active movements

The patient lifts her extended arms to shoulder height and then drops them down. Then she lifts her extended arms toward the ceiling and drops them down. The arm drops are repeated a couple of times. Next, she swings her arms in contrary and rhythmic movements while at the same time slightly bending her knees. Following this exercise, she swings her arms symmetrically forth and back while slightly bending her knees. She is given time to figure out the moves. Finally, she performs a balance test by standing on one foot.

###### Sequence three: sitting and active movements lying down

In the third sequence, the patient sits as comfortably as possible on a stool before she gets up and lies down on the floor in a comfortable position on her back. Then she flexes her knees with her feet flat on the floor, lifting her pelvis, and lowering it again after five seconds. The last task is to lift her straight arms up and place them over her head, resting there for a short while before lifting them back and place them alongside of the body.

#### Audio recordings

After the video recording of the post-treatment test, the first author/PT and the patient watched the film clips of the movement test together. Their conversation while comparing the performances of the test pre- and post-treatment was audio recorded and constitutes the interview data in this study. The patients watched the whole video material of themselves. The first author/PT played off a sequence from the pre-treatment test and then the corresponding sequence from the post-treatment test. She informed the patients that she was not looking for specific answers. Rather, she emphasized her interest in hearing the patients’ reflections on what they saw and possible changes in their movement quality and what they associated these changes with. Questions guiding the conversations were: “Did you experience doing the test different this second time?,” “What do you think when you see yourself?” and “Do you experience the difference you see, in your daily life?.” Apart from these questions the conversations were mainly derived from the video recordings they were watching. The patients were encouraged to talk freely during the playback and afterwards. The conversation was recorded and lasted for about 1 hour.

### Analysis

The analysis process started during the data collection, as the interaction between the patient and the first author/PT involved a dynamic and ever-developing mutual interpretation. In the following we describe the second tier (Høffding and Martiny, 2016) of this study as the analysis process while transcribing and coding the data material.

#### Transcription process

The data recordings were transcribed by the first author/PT and the transcription of the video-recordings became a vital part of the analysis process making us change our analytical focus during the process. The first author/PT started out by describing with words the anatomical prominent changes in movements (in the different tasks) between the pre- and post-treatment testing. However, she became aware of details in the participants` movements (behavior) and expanded the descriptions with details like *scratching her back* or *adjusting her clothes,* which again drew the attention to expressions in their way of moving, like *being hasty* or *walking self-confident*. The transcript of the video-recordings ended up consisting of the descriptions of the patients’ movement quality (including bodily expressions), and a verbatim transcription of the verbal conversation between the first author/PT and the patient during the video recording. In the process of transcribing, the first author slowly began to see the test situation more as an interaction between the first author/PT and the patient than a representation of the movement quality of the patient at two given times. The transcription of the audio recordings additionally revealed the in-depth interviews to be an interaction between the patients and the first author/PT. Her profession made her act out as a physiotherapist, sharing her expertise about the patients’ movement quality during their conversations while at the same time carefully considering the patients` reactions.

Understanding the data material as the result of the interaction between the patients and the first author/PT made us turn to enactive theory in the analysis process. Having a common source in the concept of embodiment, both the enactive theory and phenomenology served as a theoretical lens which made us realize that the Standard Mensendieck test revealed more information than the test is intended to score. Seeing the test situation as an interaction gave evidence to the overall impression of the test situation to be challenging for the patients. Additionally, the video recordings showed the participants’ bodily transformations from the pre- to post-treatment testing.

#### Coding process

The coding process was done in several steps. Initially the transcripts from the video and the audio recordings were coded individually. The audio transcripts were coded including the context of the first author/PT’s input and responses to the patients’ statements.

As a first step in the coding process of the video transcripts the first author wrote a summary of the overall impression of each patient’s performance of each task, from both the pre- and post-treatment tasks. In this process she found some initial codes describing each patient’s movement patterns and changes after treatment. This initial coding then opened for increasingly nuanced codes while returning to the full transcripts, coding both similarities and differences in the patients’ movements seen together.

When categorizing the codes, it became clear that the codes from the audio and video transcriptions somehow overlapped. Accordingly, in the final step both audio and video data were included in the same coding material and then categorized in search for patterns between the participants as well as between their expressions (during the video-recordings) and their reflections. The software program NVivo12 was a help in the structuring of the overall material.

All three authors watched the film recordings, listened to the interviews, and read the transcripts to gain a sense of the material. They continually evaluated and discussed the essence of the material, and the analysis continued throughout the writing process ending with the following two themes: *A challenging test situation* and *Development of mutual understanding.* These themes, emerging from the performances of the Standard Mensendieck test (and from the conversations when watching the performances) show to another understanding of movements than the test is intended to score. We found the test useful to show changes in movement quality and including the “expressive body” in our interpretation expanded our understanding of the patient.

In the results section, we illustrate the presentation of the themes with excerpts from the video transcripts (marked as Video) and quotes from the audio transcripts (marked with Audio). In the excerpts from the video transcripts, the verbal expressions are marked with bold font. To illustrate the simultaneity of talk and movement, we use the sign /. In the audio excerpts, the sign (…) refers to omitted words and sequences that shorten the quote. The first author is termed PT and the patients are given fictive names.

### Findings

#### A challenging test situation

The analysis shows that the testing situation was demanding for the patients. Despite discomfort like pain and awkwardness, the patients strived to fulfill the tasks. Pain expressions were observed in most of the patients through small and large bodily signs, infrequently followed by words. By observing increased tension, withheld breath, blinking, grimaces, and compensatory movements, the PT detected their pain, but when she asked, they downplayed the importance with statements like “it’s going well” and “there’s nothing dangerously painful.” This double communication, where the patients say it is ok while their bodily expressions show the opposite, was most evident in relation to pain. Eva struggled with the balance test. While standing on her right leg lifting her left foot barely off the floor, the PT asked if she was able to lift her leg higher:

Video: Eva pulls her left leg up to 90 degrees and “flies” with her arms / **PT: yes, and yes and 3, 2, 1** (counting down) **yes, that’s it**/ Eva leans to the right and the inside of her foot and big toe lose contact with the floor, her entire body trembles, and wobbles. **PT: that’s it, great!** Eva puts her leg down and rubs her right thigh / **Eva: I struggled a lot more. PT: yes, did you feel there was a difference between the sides? Eva: uhm, I felt it all the way down my leg, it didn’t feel good** / She rubs all the way down her leg before straightening up, lifting her elbow toward the ceiling, and scratching the top of her back / **PT: it’s not supposed to hurt. Eva: no, but it’s just that my body…** / She extends her arms in frustration and turns to profile. **PT: so, what you’re saying is that you have a lot of problems. Eva: yes**

Emphasizing the interaction between the patient and the PT, the PT responded to what she perceived as a vulnerable situation with positive feedback. She gave approving statements when the task was completed and directed their attention to the fact that Eva stood steadier on her left leg. As a response, Eva referred to pain in her leg as a reason for her struggling, underlining the painful sensation by stroking down her leg and referring to her body with resignation in both word and gesture. Eva fulfilled the whole test regardless of pain during several tasks. After completing the pre-treatment test, she ended up crying in pain in a fetal position on the floor. The PT was able to calm her down after a while, and Eva was the only one pushing herself this far.

Besides pain, the patients expressed unease through grimaces, autonomic reactions, restless movements, scratching themselves, and “fumbling” with their clothes and hair. In the following excerpt, Chris struggled with the task of swinging her arms in a contrary motion while slightly bending her knees. Because she kept her legs motionless, the PT asked her to include her legs in the movement:

Video: She looks hesitantly at the PT and down at her legs while she continues swinging her arms. **PT: so, you bend and stretch** / (demonstrating the movements). **Chris: Oh** / smiles slightly and starts bending her knees in time with the swing twice before she loses her rhythm the third time, stops, smiles (and “snorts” as she laughs) while bringing her arms up to her head and swirling her hands. **Chris: aah, coordination!** / laughing. **PT: Yes! Just try a bit more, see if you can give yourself some time to get into it**. Chris smiles exasperatedly and furrows her brows as she tries. She blushes faintly. Her arms are swinging but she cannot coordinate her legs and arms and her knees tentatively bend out of step with her arms. She almost stops completely. **Chris: I don’t understand** / shakes her head but continues to try and eventually finds the rhythm. She grimaces exasperatedly and loses the energy in the movements, carefully swinging her arms and bending her knees. **Chris: Like this? PT: Yes, great! Great!** Chris stops and smiles while scratching behind her ear, watching the PT.

Through complex bodily expressions, Chris revealed her awkwardness. Her gestures and blushing displayed her feelings of not mastering the coordination task, although she kept on trying. The PT tried to help with a second description and demonstration, as well as encouraging Chris to try again. Chris took the call, but although she finally cracked the code and coordinated her arms and legs rhythmically, her grimaces, sudden loss of energy and restless scratching indicated embarrassment. Chris seemed very uncomfortable throughout the whole test, despite the PT’s effort to lift the situation by keeping a light and easy tone and encouraging her to imagine being somewhere else.

Although the patients seldom verbally revealed their discomfort, the PT responded to their expressions of unease. To take the sting out of being observed, she used humor and especially avoided silence in the tasks where the patients were observed without moving. The PT tried to help by pronouncing words rhythmically when they expressed helplessness during the active, swinging movements. While observing their stiffness, she would invite more natural movements by encouraging them to feel heaviness in their bodies or pretend the camera was not there. The PT’s efforts to make the situation as comfortable as possible sometimes worked, though not always.

When watching themselves in the video replay, the patients highlighted that it was challenging to be tested and video recorded and that the unnatural test situation affected their quality of movement. In watching herself Felicia explained how her body expressed unease:

Audio: I’m a bit cautious, but maybe it’s a bit like that when you have to move slowly in front of someone, like on camera, I don’t know. My upper body is very rigid (laughing loudly) (…) but my legs just hurry along. And I keep my hands fairly rigid. No, it just looked (…) unnatural.

The video clips sometimes capture the patients’ remarkable change in their way of moving when considering themselves having fulfilled the task. For example, Ann gave the impression of relief when ending the posture observation task:

Video: She laughs and nods, loosens her posture, and walks toward the camera while smiling and pulling on her ponytail.

The way Ann walked after finishing the task was looser and freer than the way she walked during the test.

Hurriedness was a common expression among the patients. Rather than staying focused on the present task, they repeatedly asked questions while moving: *Should I do it again? Should I do sideways too? Am I done now?* Their focus on the next task affected the performance of the present task, like leaving no time to let gravity move their arms, or letting the arm movements stop by themselves.

The patients explained their bodily expressions to reflect their emotional life and emphasized that they appeared less stressed post-treatment. Some of the patients indicated that it was easier doing the test post-treatment because they knew the test and the test situation better the second time, while others expressed greater confidence in themselves in general. Most of them were hasty and performed almost no adjustments ahead of the task in the pre-treatment test, whereas post-treatment they demonstrated enhanced self-awareness through extensive preparation ahead of the tasks and fine adjustments during the tasks. Felicia reflected around her expression of increased calmness:

Audio*: yes, (laughs) I don’t know, it might be about confidence. If I’m at home, I might not have any issue dancing in front of a mirror, but when I’m in front of others I think “Oh no”! I can’t do this! I just end up becoming a bit stiff*.

Felicia revealed her movements to reflect the situation as safe or unsafe.

Watching herself in the video replay of her post-treatment test, Susan referred to a particularly large difference in the reduction of her constantly restless movements:

Audio: *The first time, I was very concerned with doing it right and maintaining some control to ensure that it was completely right, but I was a lot more relaxed here. You can see that I was (…) I’m actually able to let go of some of that control now. I feel a bit freer*.

Susan related her previous restless movements to her concerns with doing the right thing. Most participants were fumbling much less post-treatment, indicating increased calm and less discomfort during the post-treatment test.

#### Development of mutual understanding

While in the interview section, watching and talking about the video clips the PT was more or less active in the conversation depending on the patients feed-back. The PT barely shared her own reflections about changes in the patients` movement quality when the patients expressed self-assurance. This was the case in the conversation with Ann, who with a clear and steady voice articulated the differences she saw and felt in her way of moving:

Audio: ***Ann:** I seemed a lot more tense in the previous video. I look calmer now and I notice that when I’m exercising, that it feels easier than before. My body feels looser, and I breathe better. I don’t look as rigid. I feel that I rest better when I lie down and that my arms are much looser. Abdominal movements too, I breathe properly*.

On the other hand, when the patients expressed insecurity by body language, hesitant considerations, stuttering, unclear descriptions, mumbling, and whispering, the PT shared her views to a larger extent. Some frequently asked the PT what changes she saw in the recordings, or replied: *I do not know if I see any difference* and *it is difficult to explain what I see*. Susan had trouble explaining the transformation she saw in her way of walking:

Audio: ***Susan****: The first video seemed a lot more stressful, it looks like I’m doing it differently, but I don’t know*.

**PT**: (…) Earlier you said that you stand on your toes, notice how you walk without putting weight on your feet. (…) It’s almost as though you do not wish to leave any tracks (in the snow).

**Susan**: Oh yes, I see what you mean. I can actually see it, and that might be why it looks like I’m much more stressed.

The PT reminded Susan about her earlier statement and guided her attention toward how she in the pre-treatment test did not allow for gravity’s role while walking. By making such remarks the PT meant to help the patients put into words what their bodily changes expressed, or to assist them in revealing what meaning they put into the changes. Tania was surprised and excited about having a more open appearance, but had trouble putting her thoughts about bodily attitudes into words:

*Audio:* ***Tania:*** *If you walk and stretch backwards a bit, you almost end up feeling a bit gratified (laughs). And I felt a bit more closed up when leaning forwards*.

**PT**: Yes, OK. You pull yourself together a bit (referencing Tania’s body language during their chat). And you referred to it as gratifying when you open your chest - and this was something you recognized in yourself?

**Tania**: Yes. I might not have felt quite as unhinged, but it will be interesting to see because earlier I felt that I looked that way.

**PT**: Ok, what do you think now, when you can see yourself walking?

**Tania:** Hmm, perhaps not quite as far forward with the shoulders?

**PT**: No! A bit more open in the chest area?

**Tania:** I think I don’t feel that I’m bending as far forward.

**PT:** Yes, I thought the difference was quite significant, didn’t you?

**Tania**: (laughs) Yes, I did actually. I find it surprising, but the thing about leaning forwards (…) was a bit fun to see (…) my attitude is much better (laughs).

The PT picked up on Tania’s uncertainty and tried to help her by being curious and following up on her demonstration of a contracted body. Now and then the PT’s questions invited the patients to connect their quality of movements to expressions of their emotional life. Susan and the PT observed major changes in Susan’s appearance. She seemed more relaxed and flexible and showed an increased ability to drop her arms freely:

Audio: **PT:** So now you’re able to let go. But have you let go of anything?

**Susan:** (slight laughter) Do you mean letting go in a physical sense?

**PT:** Perhaps, (…) we say, “to let go” and “to cling onto” things and we can cling onto something physical or non-physical. Letting go could refer to something physical or not.

**Susan:** Yes, but as I said earlier as well (…) I know that I experience a general sense of feeling more relaxed.

**PT**: You mentioned control, letting go of control.

**Susan**: Yes, letting go of control, I work on letting go of things day-to-day, making changes. That’s also about letting go of something.

The PT encouraged Susan to reflect on a connection between changes in her way of moving and changes in her daily life. Helping the patients to interpret their bodily changes made them reflect on their own bodily mechanisms. Susan further elaborated on how she had started to let go of her stringent lifestyle as a way to take better care of herself.

Watching the video clips made the patients reflect on how their bodily transformations had come about. They justified their new appearance with treatment making them aware of their own bodily mechanisms and their possibility to behave in a less tense way. The patients felt that stress and insecurity still affected them physically by making them tense, which led to pain. However, they better tolerated and accepted these feelings, and could even use them as a way of working with themselves. Felicia explained how she physically practiced making herself stand out more:

Audio: *I felt a bit tense when I sat down* (at a shoe shop) *but I told myself (her voice becomes louder, clearer, and faster) “No! I can do this.” and then I put my feet down like this (she stomps her legs down onto the floor) (…) I’ve thought about it on the bus too, that I often sit like this (gathers and tightens her legs). I now make sure to sit the way boys sit, with my legs out. I’m just going to take up some space now, right? I’ve thought about confidence and self-esteem a lot too. I often come across as “apologizing for existing” (she puts on a squeaky voice and makes herself look smaller) and end up sitting like this. So, I’m trying to remember that the more relaxed I sit, the better I will feel. Maybe it makes you feel a bit more confident, what do you think?*

Felicia added that listening to and expressing her own needs took practice and sometimes felt uncomfortable. The patients emphasized that having become more secure was evident in the quality of their movements. Susan pointed out that the pre-treatment test was recorded when she was in a particularly “stormy” life situation, and that she was more present in her body in the post-treatment recording. When witnessing her improved balance, Eva told how this had come about without her noticing. Reflecting on her bodily changes of decreased tension and increased flexibility, she said that she felt more confident in her body, realizing that she had additionally increased confidence in her boyfriend. In this way, the patients underscored that their bodily transformations could depend on changes in their life circumstances, but that it may well be the other way around: a changed body could change their experience of themselves and their life circumstances.

The patients also emphasized the value of seeing the changes in the video clips. From a physiotherapists point of view the patients were more relaxed post-treatment and overall revealed an increased ability to give in to the force of gravity in all positions, as well as to let the arms fall in a relaxed drop. They walked more freely and showed increased symmetry as well as changes in the alignment and the mobility of their back, pelvis, and hips. Coordination was improved and they adjusted the use of force by letting gravity act on rhythmic movements. Increased stability was evident in both the balance exercise and the pelvic lift. The decrease of tension in their abdomen, neck and shoulders related to their more open chests and eased breathing. For Melanie, seeing the changes fortified her recovery process:

Audio: *Yes, I’m quite surprised (…). I thought you might be able to see a difference using more advanced methods, but you can actually see it pretty clearly. So, it’s fun to see a conclusion in the video. Sometimes I find myself wondering whether I just imagine the changes. Like I said, when things happen slowly and you are involved yourself, then it’s not always so easy to realize. It’s encouraging because I sometimes feel frustrated.*

Watching and elaborating on the video recordings gave valuable information to both the PT and the patients. Having made sense of their bodily transformations, some of the happy and proud patients asked if they could have a copy of the video recordings.

## Discussion

Our analyses revealed that the performance test Standard Mensendieck test can provide information that goes beyond objective measurements of patients’ movement quality based on functional anatomy. When the first author/PT took the patients’ embodied presence into account, the subjective, sensitive, and expressive body expanded the understanding of the patient. The embodied interaction processes were important for what emerged between the patient and the first author/PT and moved the individuals’ acting, understanding and sensemaking of themselves and the situation.

The main insight from the test situation is associated with the embodied interaction, as a dynamical coupling and coordination appeared between the first author/PT and the patient. Even though the patient during the performance of the test was the one following the lead of the PT, they both actively participated in the generation of meaning through their bodily and verbal expressions. From the first author/PT’s point of view the patients` bodily expressions and intentions were perceptible and lead to an increased understanding of the patients and the situations as the patients bodily and verbal expressions sometimes were in contradiction. Even though the patients said they were ok, the first author/PT found the patients’ emotional states distinctive in their acting shown as tension, hurriedness, restless fumbling, and autonomic reactions. The lived body always simultaneously expresses a physiological base and lived experiences (Merleau-Ponty, 2002). This is an ambiguity that was part of the patient’s bodily expression in the test situation, in which the first author/PT responded by verbal and bodily feedback to support the patient in carrying out the task, while the situation also contributed to the patient vulnerability. Always situated and in constant interaction with its environments, the body is in some sense the reflection of the environment (Merleau-Ponty, 2002; Gallagher and Zahavi, 2012) and will always be characterized within a specified context, which in this situation was being evaluated and filmed when performing the test.

### Sensitive concern for the patient

Evaluating movements and posture without considering the emotional expressions reflecting the living bodies’ constant engagement with the environment, may confuse the understanding of the patient. Research shows that women with CPP often feel ignored and misinterpreted in the healthcare system (Grace and MacBride-Stewart, 2007; Shallcross et al., 2018) and our results indicate the importance of being sensitive to the patient with CPP’s embodied expressions and the situation she is put in. In the clinical setting, the health personnel have the authority, and thus the responsibility to maintain the patient’s integrity and autonomy (Pellegrino, 1990). Enactive theory comprises in the principle of autonomy an agent sustaining the capability of interaction with others and the environment (De Jaegher and Di Paolo, 2007; Sørvoll et al., 2022). When using tests, health personnel risk reducing the patient to a passive recipient of expert assessments based on predetermined criteria (Anjum et al., 2020) and thereby diminish her autonomy. Encompassing the patients non-verbal communication and including her experiences of the vulnerable test situation (and herself) may therefore prevent the patient from an experience of being treated as an object, exposed to the gaze of others (Leder, 1990; Bjorbækmo and Engelsrud, 2011). The patient’s experience may incorporate lasting effects, which will determine how she perceives and understands future experiences and herself (Bjorbækmo and Engelsrud, 2011).

### Exploring bodily expressions

The patients in our study showed major changes in their movement quality between pre- and post-treatment testing. While the patients and the first author/PT together watched the Standard Mensendieck test recordings, they explored the meaning of bodily changes and expressions (movements, autonomic reactions, voice, facial expressions) which provided further understanding and gave new insights for them both regarding the patient’s complex symptoms. As mentioned previously, intentions are expressed in action and can be perceptible to others (Merleau-Ponty, 2002; Fuchs and De Jaegher, 2009). When the patients saw their way of moving in the recordings, they pointed to their expressions as disclosing thoughts and feelings that went beyond the actual test situation, feeling unsafe and needing control in their daily life. This illustrates the ambiguity of the body, indicating the importance of being open to multiple interpretations for adequate patient care. As an example, observing their tense gait with reduced rotation in the pelvis and back while walking could be interpreted as a pure physical restriction or as a “guarded behavior” related to pain in the pelvis. In line with previous research (Boge-Olsnes et al., 2022a, b), when watching the videos of the Standard Mensendieck test our participants clarified their tense gait to be an expression of a “guarding behavior” to protect them against the feeling of vulnerability in a challenging situation, or a feeling of vulnerability based on previous experiences. This is in line with the embodied understanding of how the living body simultaneously inhabits (and expresses) past as well as present experiences (Merleau-Ponty, 2002).

The enactive approach recognizes social understanding as deeply embodied and emphasizes how we mutually coordinate by regulating behavior toward each other (De Jaegher and Di Paolo, 2007; Fuchs and De Jaegher, 2009). Thus, through the first author/PT and the patient’s interaction, while watching the video clips, the first author/PT changed her involvement in the conversations depending on the patient’s vocal and bodily expressions. In accordance with Fuchs and De Jaeger (2009) reasoning, mutual affection opened up new domains of sense-making in a process of intersubjectivity between the patients and the first author/PT through their interaction. The patient’s association between their bodily expressions and how they coped with daily life possibly reflects the first author/PT’s opening for such a perspective - somewhat guiding their attention to these dimensions of the body. In return, the first author/PT learned about how stress and insecurity in the patient’s daily life led to pain and how the patients used this insight in their recovery processes. Initiated by their expressive bodies, the patients revealed how they made use of the experiences of their bodily reactions to guide their actions, generate confidence and restore health. The Standard Mensendieck test thus proved to be a basis for further understanding for both the patient and the first author/PT. In accordance with Di Paolo et al. (2018), the patients saw their own body as something far more than an organism with pre-specified functions like a machine, but something flexible and changing in relation to both the outer and the inner world. Exchanging information and creating meaning together, is shown to create a basis for therapeutic alliance (Fougner and Haugstad, 2015; Danielsen et al., 2018; Søndenå et al., 2020). Making the body the pivot point in communication may help health personnel both to understand the patient “here and now” in the clinical setting, as well as to explore complex symptoms in collaboration with the patient. We found that watching themselves and elaborating on their bodily transformations with an expanded view on the body - considered both as an expressive field of emotions and as functional anatomy - made our patients proud and reinforced their belief in themselves and their recovery.

### To sum up

An expanded embodied understanding of the performance of the Standard Mensendieck test proved to be useful as a therapeutic mediator. We suggest that for the PT, *seeing the “whole” person* is important to gain a more complete understanding of the patient’s symptoms and challenges and for looking after the patient in a vulnerable situation by ensuring her autonomy in the interaction. For the patient, the actual interaction processes between the therapist and the patient are important to assert her autonomy. Applying the patient’s expertise on herself and her life together with the professional expertise may reinforce the identity of the patient and make health care an interdependent practice where sensemaking is a co-construction of meaning between the patient and the health personnel.

### Ethical and methodological considerations

The first author’s/PT’s use of self as an instrument for the data collection has implications for this study, as her professional expertise structured the encounters in specific ways by guiding her participation in the knowledge generation process. Furthermore, in the interaction there is an asymmetry between the persons conventionally involved in the relation between a patient (seeking help) and the PT (providing the help). However, our participants/patients were not the patients of the first author/PT, implying that they could speak freely without it affecting their treatment process. Our data are collected in accordance with the Recommendations for the Protection of Research Participants (ICMJE).

We have strived to enhance the transparency of this study by presenting the theoretical framework underpinning the research project, as well as the authors’ background (Stige et al., 2009). To provide trustworthiness, we present the results illustrated with extensive quotes and excerpts enabling the first- and second person perspective of the participants to be visible (Brinkmann and Kvale, 2015). That the first author collected the data gives the advantage insider knowledge of a wider context. Simultaneously, it presents challenges in taking an analytical distance, making the second and third author’s external views essential in the effort to be critically self-reflective about preconceptions and relational dynamics during the research process.

Our small sample has made a thorough in-depth analysis of the video recordings and the interviews possible. Although our findings are specific to our participants, their vulnerability may be transferable to women in similar situations, and the results may be relevant to a variety of health care professions using tests.

## Data availability statement

The datasets presented in this article are not readily available because this article is based on film recordings of the participants performing a test. The partcipants have in their written consent only approved for the researcher and her supervisors (the article authors) to see the film recordings. Requests to access the datasets should be directed to cathrine.boge-olsnes@uit.no.

## Ethics statement

The studies involving human participants were reviewed and approved by The Regional Ethical Committee REK Nord in January 2019 (approval no. REK-Nord 2018/2533). The patients/participants provided their written informed consent to participate in this study.

## Author contributions

CB-O had overall responsibility for the design and the conduct of the research project and the writing of the article. All authors contributed to the article and approved the submitted version.

## Conflict of interest

The authors declare that the research was conducted in the absence of any commercial or financial relationships that could be construed as a potential conflict of interest.

## Publisher’s note

All claims expressed in this article are solely those of the authors and do not necessarily represent those of their affiliated organizations, or those of the publisher, the editors and the reviewers. Any product that may be evaluated in this article, or claim that may be made by its manufacturer, is not guaranteed or endorsed by the publisher.
