# Supplementary material for: Exploring the potential of a standardized test in physiotherapy: making emotion, embodiment, and therapeutic alliance count for women with chronic pelvic pain
Source: Front Psychol. 2023 Aug 3;14:1166496. doi: 10.3389/fpsyg.2023.1166496 (PMC10437049; doi:10.3389/fpsyg.2023.1166496)
Supplement: Supplementary file 1 [file Data_Sheet_1.pdf]

## Excerpt from the manual for the Mensendieck test

The complete manual is to be found in the appendix in the article by Haugstad et al (2006). It includes a detailed description of the ideal atomical conditions in the various test tasks. Each task is subdivided into measurable joint positions or detailed descriptions of the individual role of body parts. Further, the manual describes in detail which criteria must be met to score each body part on a scale from 0-7. We present here an extract from the manual and have chosen to show the various subdivisions under *Standing posture* to illustrate the characteristics of the test manual.

## Standing posture

### a) Global/line of gravity

i) *Description*: "The body is built around an *imaginary line of gravity* that passes through the foot just in front of the ankle joint, from the mastoid process, through the major trochanter, and just in front of the lateral malleolus" (translated from Kendall, Creary, and Provance, Citation1993). "At the ankle,….the line of gravity passes approximately through the apex of the arch, designated laterally by the calcaneo-cuboid joint" (Mensendieck, Citation1937).

(1) **7-6 points**: The subject stands in the optimal line of gravity. There should be symmetry between the two sides of the body, and there is balance between agonists and antagonists at the front and the back of the body.

(2) **5-4 points**: The subject stands *slightly in front of or behind* the optimal line of gravity. There may be a *slight asymmetry* between the two sides of the body, and there may be a *slight imbalance* between agonists and antagonists at the front and the back of the body.

(3) **3-2 points**: The subject stands *markedly in front of or behind* the optimal line of gravity. There is a *marked asymmetry* between the two sides of the body, and there is a *marked imbalance* between agonists and antagonists at the front and the back of the body.

(4) **1-0 point:** The subject stands *very markedly in front of or behind* the optimal line of gravity. There is a *very marked asymmetry* between the two sides of the body, and there is *a very marked imbalance* between agonists and antagonists at the front and the back of the body.

### b) Position of the ankle

i) *Description*: "Dorsiflexion at the ankle with the knee straight is normally about 10°. This means that standing barefoot with feet nearly parallel the lower leg does not sway forward more that about 10°" (Kendall, Creary, and Provance, Citation1993).

(1) **7-6 points**: The position of the ankle is optimal as described above. The feet are parallel, and tibia is approximately 10° anterior to the vertical position on talus.

(2) **5-4 points**: The position of the ankle is *slightly* in *valgus* or *varus*, or has a *slightly* greater or less angle of flexion than 10°.

(3) **3-2 points**: The position of the ankle is *markedly* in *valgus or varus*, or has a *markedly* greater or less angle of flexion than 10°.

(4) **1-0 point**: The position of the ankle is *very markedly* in *valgus or varus*, or has *a very markedly* greater or less angle of flexion than 10°.

### c) Position of the knee joint

i) *Description*: “When we stand up, the face of the joints on the tibial condyles are approximately in the horizontal plane, and the shaft of femur makes an open angle of approximately 174° to the lateral side with the longitudinal axis of tibia. This physiological angle of abduction is normally smaller in females than in males” (Dahl and Rinvik, Citation1996).

(1) **7-6 points**: The position of the knee is optimal as described above with the longitudinal axes of the femur and tibia forming a laterally open angle of approximately 170°. The knee should be fully extended ant the patella free.

(2) **5-4 points:** The angle of abduction is *slightly larger or smaller* than approximately 170°, or the knee is *slightly flexed or extended*.

(3) **3-2 points:** The angle of abduction is *markedly larger or smaller* than approximately 170°, or the knee is *markedly flexed or extended*.

(4) **1-0 point:** The angle of abduction is *very markedly larger or smaller* than approximately 170°, or the knee is *very markedly flexed or extended*.

### d) Position of the pelvis.

i) *Description*: “In the normally aligned pelvis, the anterior superior iliac spines of the pelvis lie on a horizontal line with the posterior superior iliac spines and on a vertical line with the symphysis pubis (Norkin and Levangie, Citation1992).

(1) **7-6 points:** The position of the pelvic is optimal as described above if the anterior superior iliac spines of the pelvis lie on a horizontal line with the posterior superior iliac spines on a vertical line with the symphysis pubis.

(2) **5-4 points:** The position of the pelvis is *slightly antevert or retrovert* compared to the optimal position.

(3) **3-2 points:** The position of the pelvis is *markedly antevert or retrovert* compared to the optimal position.

(4) **1-0 point**: The position of the pelvis is *very markedly antevert or retrovert* compared to the optimal position.

### e) Position of the spinal column (back)

i) *Description*: “In the standing position, the spinal column is curved with an anterior cervical and lumbar convexity (lordosis), and a posterior thoracic and pelvic convexity (kyphosis)” (Norkin and Levangie, Citation1992).

(1) **7-6 points**: The position of the spinal column is optimal as described above with an anterior cervical and lumbar convexity and a posterior thoracic and pelvic convexity.

(2) **5-4 points**: Either of the cervical or lumbar lordosis or the thoracic kyposis is *slightly* increased or decreased, or the column has a *slight lateral* deviation.

(3) **3-2 points**: Either of the cervical or lumbar lordosis or the thoracic kyposis is *markedly* increased or decreased, or the column has a *marked* lateral deviation.

(4) **1-0 point**: Either of the cervical or lumbar lordosis or the thoracic kyposis is *very markedly* increased or decreased, or the column has a *very marked* lateral deviation.

### f) Position of the shoulder

i) *Description*: "Normally, the scapula is said to rest at a position on the posterior thorax approximately 2 in from the midline, between the second through seventh ribs" (Norkin and Levangie, Citation1992).

(1) **7-6 points:** The position of the shoulder is optimal as described above with the scapula rest at the position on the posterior thorax approximately 2 inches from the midline, between the second through seventh ribs. The clavicle should rest on the first rib.

(2) **5-4 points**: The inferior angle of the scapular wing a *slight* distance from the thoracic wall, or the humeral head is drawn *slightly* forward or upward, compared to optimal position.

(3) **3-2 points**: The inferior angle of the scapular wing has a *marked* distance from the thoracic wall, or the humeral head is drawn *markedly* forward or upward, compared to optimal position.

(4) **1-0 point**: The inferior angle of the scapular wing has a *very marked* distance from the thoracic wall, or the humeral head is *very markedly* forward or upward, compare to optimal position.

### g) Position of the neck

i) *Description*: "The line of gravity relative to the head passes through the external auditory meatus posterior to the coronal suture and through the odontoid process. The line of gravity falls anterior to the transverse axis of rotation for flexion and extension of the head and creates a flexion moment. The gravitational moment, which tends to tilt the head forward, is counteracted by tension in the ligamentum nuchae and tectorial membrane, and by activity of the neck extensors. When a postural analysis is being performed, the gravity line should pass though the lobe of the ear" (Norkin and Levangie, Citation1992, p. 431–432).

(1) **7-6 points**: The position of the head is in the midline, with a cervical lordosis.

(2) **5-4 points**: The head is *slightly* out of position in the frontal plane or for rotation, or there is a *slightly* increased or diminished cervical lordosis.

(3) **3-2 points:** The head is *markedly* out of position in the frontal plane or for rotation, or there is a *markedly* increased or diminished cervical lordosis.

(4) **1-0 point:** The head is *very markedly* out of position in the frontal plane or for rotation, or there is a *very markedly* increased or diminished cervical lordosis.
